# Troubling bonds: lipid unsaturation promotes selenium dependency and sensitivity to ferroptosis

**DOI:** 10.1038/s44321-024-00150-x

**Published:** 2024-10-07

**Authors:** Ancély Ferreira dos Santos, José Pedro Friedmann-Angeli

**Affiliations:** Rudolf Virchow Center for Integrative and Translational Bioimaging, University of Würzburg, Würzburg, Germany

**Keywords:** Autophagy & Cell Death, Cancer, Metabolism

## Abstract

J. P. Friedmann-Angeli and A. F. dos Santos highlight two complementary studies recently published in *EMBO Mol. Med*. reporting examples of alterations in lipid metabolism that can promote targetable vulnerabilities for breast tumors with poor prognosis.

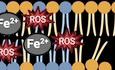

## Teaser

Cancer cells exhibit a remarkable ability to orchestrate metabolic adaptations to survive and proliferate. Such metabolic adaptations, including carbohydrates, amino acids and lipid metabolism, have been shown to also contribute to the pathological cascade during cancer progression and therapy resistance. Importantly, these adaptations provide a rational for targeted therapies inducing selective cell death. Recently, two studies by Lorito et al (Lorito et al, 2024) and Ackermann et al (Ackermann et al, 2024) published in *EMBO Molecular Medicine* reported examples of alterations in lipid metabolism that can promote targetable vulnerabilities for tumors with poor prognosis.

**Figure 1:**
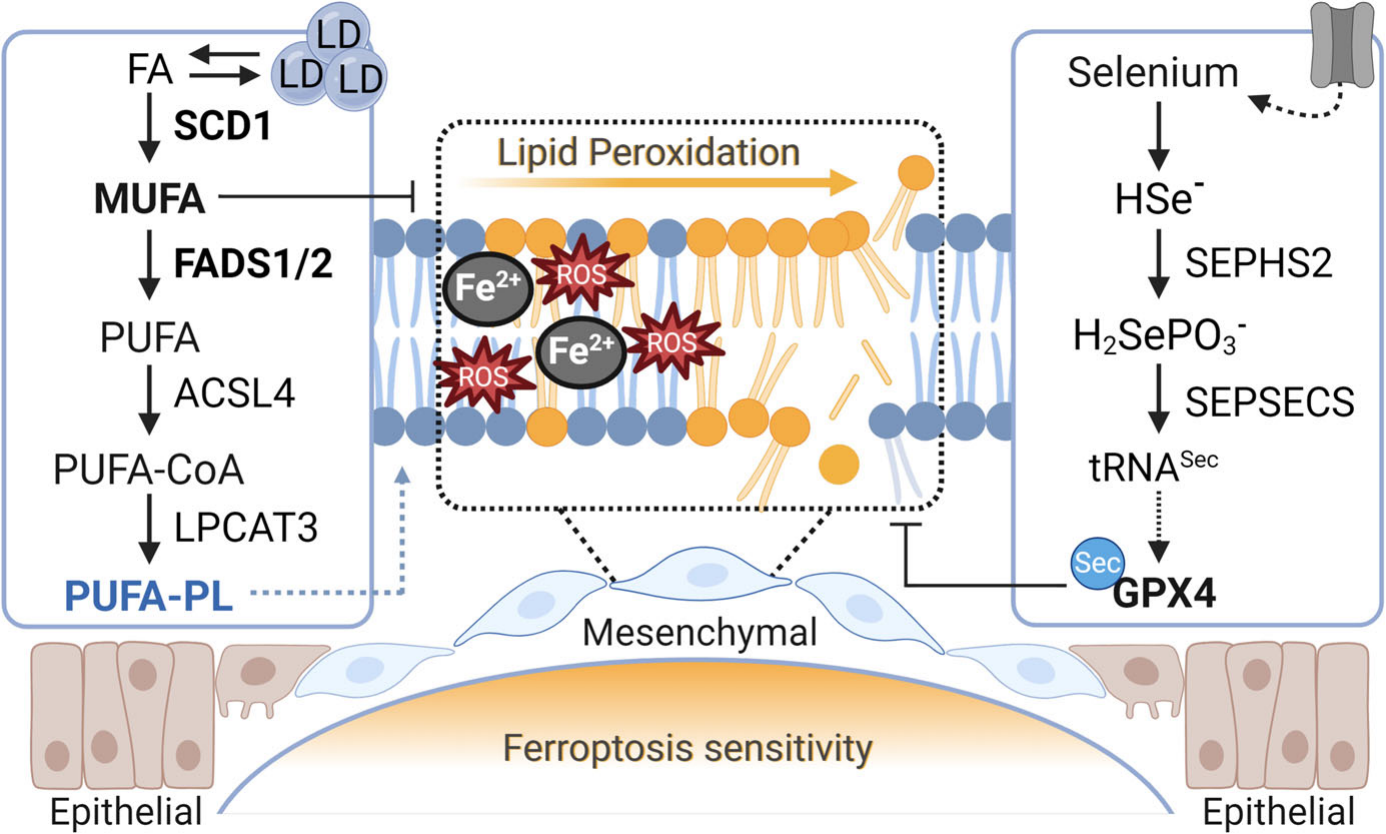
Lipid metabolism regulates cell death by ferroptosis. Reactive oxygen species (ROS), in combination with free iron (Fe^2+^), can promote the damage of polyunsaturated fatty acids (PUFAs) within phospholipids (PUFA-PL), triggering phospholipid peroxidation and ultimately leading to ferroptosis. Triple-negative breast cancer (TNBC), a highly aggressive breast cancer subtype, exhibits elevated levels of the desaturases FADS1 and FADS2, which enhance PUFA biosynthesis increasing susceptibility to ferroptosis. Increased lipid droplet (LD) formation at the primary tumor site further elevates monounsaturated fatty acids (MUFAs) levels, protecting against PUFA oxidation. In contrast, metastatic cells in a mesenchymal state heavily depend on GPX4 and the selenoprotein biosynthetic machinery to prevent phospholipid peroxidation. These insights reveal potential vulnerabilities in metastatic breast cancer that could be targeted through ferroptosis-based therapies. Blue lipids: enriched in PUFA; Yellow lipids: peroxidized; Brown cells: epithelial state; Blue cells: mesenchymal state. Figure created using BioRender.

Phospholipids are involved in essential processes, acting as an alternative energy source, precursors for secondary messengers, and impacting membrane fluidity (Martin-Perez et al, 2022). The basic structure of a phospholipid consists of a glycerol backbone linked to two fatty acid (FA) molecules and a phosphate head group. FA can be further subdivided, according to the number of double bonds in their acyl chain, as saturated, mono, or polyunsaturated FA (SFA, MUFA, or PUFA). During the biosynthesis of MUFA/PUFAs, the first double bond is inserted by the enzyme stearoyl-coenzyme A desaturase (SCD1) (Fig. 1). Subsequently, additional double bonds are incorporated by fatty acid desaturases 1 and 2 (FADS1/2). The promotion of lipid desaturation leading to PUFA accumulation was demonstrated to be involved in tumor metastasis and therapy resistance. Nonetheless, PUFA’s are notoriously prone to autoxidation, which has been associated with high sensitivity to ferroptosis, thus offering a window of opportunity to target cancer cells associated with a PUFA-high state (Vriens et al, 2019).

Ferroptosis was coined in 2012 (Dixon et al, 2012) to describe a cell death modality characterized by the iron-mediated peroxidation of lipids, specifically oxidation of PUFA-containing phospholipids. The presence of PUFAs in cellular membranes is considered essential for ferroptosis execution. The phospholipid peroxidation process promotes the accumulation of phospholipid truncated species, leading to cell swelling and eventual plasma membrane rupture (Freitas et al, 2024). Inhibition of phospholipid peroxidation and ferroptosis depends primarily on the activity of the selenoprotein glutathione peroxidase 4 (GPX4) (Fig. 1). GPX4 is essential for repairing peroxidized phospholipids by directly reducing phospholipid hydroperoxides (Ursini et al, 1982). Previously, a positive correlation was shown between the expression of acyl-CoA synthetase long-chain family member 4 (ACSL4) and ferroptosis sensitivity in a subset of breast cancer cell lines (Doll et al, 2017). ACSL4 activates the PUFA arachidonic acid (AA) to be incorporated into phospholipids. ACSL4 was highly expressed in basal-like breast cancer cell lines, enriching their membranes with AA and predisposing cells to ferroptosis. At that time, it was unclear if the effect resulted from the increased uptake of AA or if there was an active machinery associated with elongation and desaturation in these breast cancer cells. In their study, Lorito et al, uncovered that intracellular PUFA availability was increased in aggressive triple-negative breast cancer (TNBC) cells because of the metabolization of MUFA by FADS1/2 desaturases. Remarkably, they also report that ferroptotic insults promoted the modulation of intracellular lipid composition, and those adaptations were associated with a poor prognosis. Moreover, Lorito et al showed that the number of LD was reduced by FADS1/2 inhibition due to impaired PUFA formation. Interestingly, under ferroptosis stimulation, TNBC increased the number of LD, suggesting a mechanism exploited by these cells to prevent PUFA oxidation by reducing access of their double bonds to the intracellular oxidative insults. Therefore, LD metabolism could be further investigated as a novel approach to target ferroptosis-resistant cells with increased LD content.

Ackermann et al, obtained complementary results, showing that SCD-produced MUFAs were present in the conditioned medium of TNBC, associated fibroblast cells, and extracellular microenvironment. These MUFAs contributed to colony-stimulating capacity by suppressing ferroptosis, helping cells adapt and thrive under stress. These observations align with others that report the functional consequences of lipid metabolism alteration in the pathophysiological cascade of cancer development and therapy resistance. Specifically, incorporating PUFAs into phospholipids promotes membrane fluidity and cell mobility, all essential steps during epithelial-mesenchymal transition (EMT). A recent report indicates that ZEB1, a central transcription factor involved in EMT, regulates key lipogenic enzymes and promotes membrane remodeling by repressing SCD1 and activating critical enzymes involved in PUFA production (such as FADS2) and incorporation into phospholipids (ACSL4) (Schwab et al, 2024). Cells with high levels of ZEB1 are characteristic of undifferentiated carcinomas, which are regarded as being highly plastic and associated with a high metastatic capacity and drug tolerance. Meanwhile, such a “PUFAing” process discloses a cancer vulnerability to ferroptosis, and the studies from Lorito et al, and Ackermann et al, clarified aspects that could contribute to TNBC lipid remodeling that ultimately shape the response to ferroptosis. These findings reinforce the therapeutic rationale for targeting ferroptosis in this context, and the PUFA/MUFA ratio could guide ferroptosis susceptibility.

An additional observation with important implications for targeting these PUFA-high states was made by Ackermann and colleagues, who showed that ferroptosis was promoted upon selenium starvation. Sensitivity to ferroptosis is tightly related to selenium availability due to its importance for the biosynthesis of GPX4. Selenoproteins are proteins containing the amino acid selenocysteine (Sec), a structural analog of cysteine, which contains selenium instead of sulfur. Sec biosynthesis requires a Sec-specific transfer RNA (tRNA^Sec^), a defined Sec insertion sequence (SECIS) element in the 3′-untranslated region in the mRNA of a selenoprotein, and a unique riboprotein complex that recognizes and incorporates Sec into nascent polypeptides (reviewed in Dos Santos et al, 2023). This machinery depends on the availability and metabolization of the micronutrient selenium, which can be acquired from the diet in organic and inorganic forms via extracellular carrier proteins. In the liver, hepatic cells produce selenium-carrier protein selenoprotein P (SELENOP) that is used as a transporter of Sec to the extra-hepatic tissues. SELENOP uptake in target cells and tissues is mediated by its receptors, such as apolipoprotein E receptor 2 (APOER2/LDL Receptor Related Protein 8, LRP8) and megalin (LDL Receptor Related Protein 2, LRP2). LRP8 was previously identified as a specific vulnerability in ferroptosis-sensitive cancers, such as MYCN-amplified neuroblastoma cells (Alborzinia et al, 2023). Further supporting the untapped potential of targeting selenocysteine metabolism, Ackermann et al unveiled that the impairment of selenoprotein synthesis by inhibiting tRNA^Sec^ formation decreased lung metastasis of TNBC cells.

The studies from Lorito et al, and Ackermann et al, establish that alterations in lipid metabolism can reveal metabolic liabilities within a subset of breast cancers that lack effective treatment options. They suggest that the administration of exogenous PUFAs, as well as targeting SCD1 or FADS1/2 desaturases or tRNA^Sec^ synthesis, could improve cancer treatment by stimulating ferroptosis. Identification of altered mechanisms could further lead to more precise prognostic assays. Metabolic rewiring events associated with drug resistance, dedifferentiation, and proliferation in various cancer types have also been linked to increased sensitivity to lipid oxidation and the accumulation of lipid droplets (Zadoorian et al, 2023). These insights could prove crucial in understanding ferroptosis sensitivity beyond triple-negative breast cancer (TNBC).
